# Comparable outcomes with improved esthetics: da Vinci-assisted neck dissection for early stage oral cancers

**DOI:** 10.1007/s11701-025-02656-z

**Published:** 2025-08-17

**Authors:** Wen-Chun Lin, Man-Wei Hua, Chen-Chi Wang

**Affiliations:** 1 Department of Otorhinolaryngology Head and Neck Surgery, Taichung Veterans General Hospital, 1650, Section 4, Taiwan Boulevard, Xitun Dist., Taichung, 407219 Taiwan; 2 Department of Post‐Baccalaureate Medicine, College of Medicine, National Chung Hsing University, Taichung, Taiwan; 3 School of Medicine, Chung Shan Medical University, Taichung, Taiwan; 4 Institute of Clinical Medicine, National Yang Ming Chiao Tung University, Taipei, Taiwan; 5 School of Medicine, National Yang Ming Chiao Tung University, Taipei, Taiwan

**Keywords:** Robotic surgery, Neck dissection, Oral cancer, Retro-auricular

## Abstract

This study compares the retro-auricular robotic-assisted approach using two or three robotic instruments with conventional open neck dissection in patients with early N stage oral cavity cancer. Twenty-six patients with T1-3N0-1M0 disease underwent supraomohyoid neck dissection between 2018 and 2023 (13 robotic, 13 open). Perioperative outcomes, including hospital stay, drainage duration, complications, and lymph node yield, showed no significant differences (median lymph nodes: 20 vs. 21; *p* = 0.950). During a median follow-up of 743 days, no recurrences or deaths occurred. In the last five robotic cases, a third instrument (ProGrasp Forceps) was added without increasing operative time (*p* = 0.803). Robotic-assisted neck dissection via the retro-auricular route is a safe and effective alternative for early stage oral cavity cancer patients, particularly those with clinical N0 disease, preserving oncologic outcomes while improving cosmetic appearance.

## Introduction

The neck region contains abundant lymphatic circulation, making it a common pathway for the metastasis of upper aerodigestive cancer cells to cervical lymph nodes. Even in patients with early stage oral cavity cancer, who show no apparent lymphadenopathy, approximately 15–20% may have occult lymph node metastasis in the ipsilateral neck. Without dissection and pathological examination of high-risk neck lymph nodes to determine the true N-status, metastatic lymph nodes that are detectable only under a microscope can be easily overlooked [1]. This can lead to local recurrence of head and neck cancer, posing life-threatening risks.

In the past decades, patients with clinical N0 oral cavity cancers have undergone conventional open neck dissection to confirm their N-status. Approximately 70% patients of these patients were found to have pN0 results after surgery but endured visible neck scars. This issue is particularly significant for patients prone to keloids, where psychological stress related to surgical scarring often leads to fear of surgery and reduced satisfaction. Some authors have proposed prophylactic radiation therapy to the ipsilateral neck, which avoids visible scarring but introduces long-term adverse effects such as hyperpigmentation, muscle fibrosis, and carotid artery stenosis [2, 3].

Historically, head and neck surgeons have been hesitant to adopt traditional endoscopic techniques via hidden remote incision due to the complex anatomy of the region, which poses challenges in access and visualization. However, recent advancements in surgical techniques such as robot-assisted surgery have emerged to offer endoscopic approach with efficacy comparable to open surgery. Studies have demonstrated that robotic-assisted neck dissection not only achieves favorable oncological outcomes but also enhances patients’ quality of life [4–6].

This retrospective study investigates the robotic-assisted retro-auricular approach compared to the conventional open method for neck dissection in oral cavity cancer patients at a single medical center. We would like to preliminarily evaluate the feasibility of using two or three robotic instruments for supraomohyoid neck dissection regarding perioperative and oncologic outcomes.

## Materials and methods

### Patients

This retrospective analysis was conducted on data collected from patients diagnosed with oral cavity cancer without distant metastasis who underwent surgical treatment in the Otorhinolaryngology Head and Neck Department of Taichung Veterans General Hospital, Taiwan, between 2018 and 2023. The study was conducted according to the Declaration of Helsinki. The study protocol was approved by the Institutional Review Board of the TCVGH (IRB no. _CE25239B_). Patients who had clinical N3 lymph node metastasis, neck skin involvement, history of other head and neck cancers, prior radiotherapy or concurrent chemoradiation to neck, and any history of neck surgery were excluded. The collected data were analyzed descriptively to assess surgical outcomes. The primary tumor site in all cases underwent wide excision without microvascular free flap or rotational flap reconstruction. The study compared the data of 13 patients who received robotic-assisted supraomohyoid elective neck dissection via retro-auricular approach and 13 patients who underwent conventional supraomohyoid elective neck dissection with similar tumor stages. Data were compared between the two groups, including perioperative outcomes (blood loss during surgery, number of lymph nodes retrieved, duration of drainage, length of hospital stay, complications (if any), and oncological outcomes (regional neck recurrence rate, and survival rate) [7].

### Robotic technique with retro-auricular approach (RA)

**Fig. 1:**
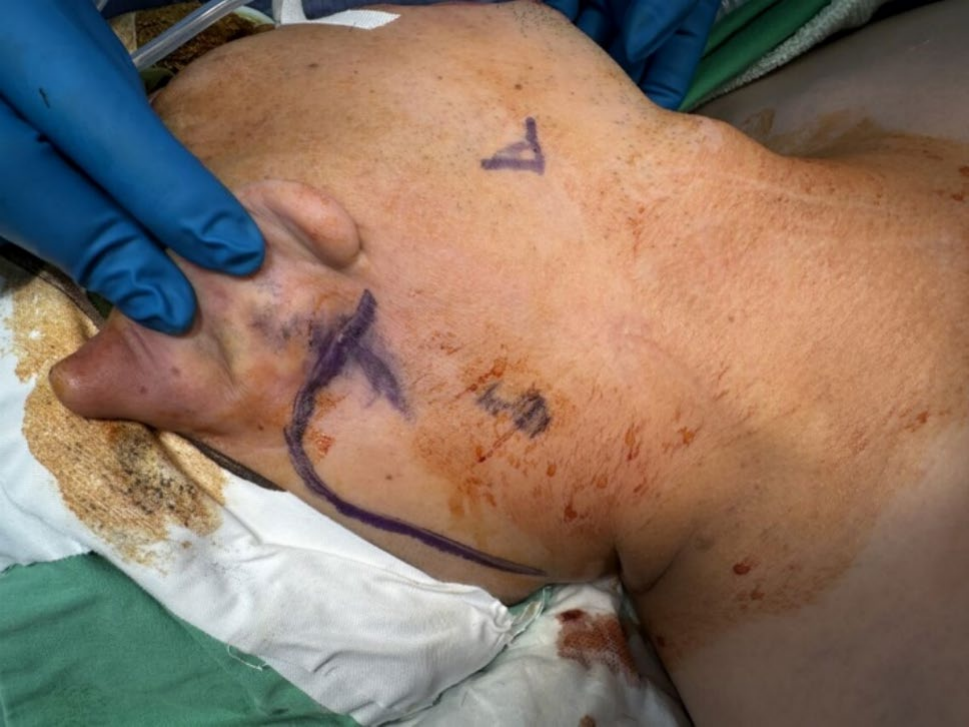
The retro-auricular incision extends superiorly from ear lobe to mid-point of auricle along the post auricular sulcus. Then incision curved down along the hair line

**Fig. 2:**
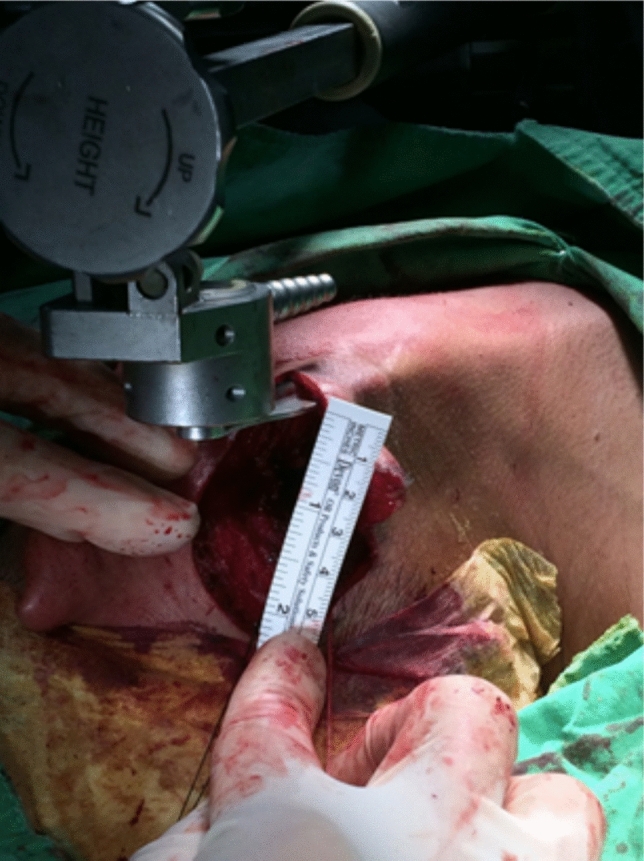
The retro-auricular skin flap was fixed with the Modena Retractor System, creating a subcutaneous surgical tunnel exposing the supraomohyoid neck level I–III

After general anesthesia, a shoulder roll was put over the patient’s shoulder to keep the neck extension. The patient was placed in a supine position with head turned to opposite side. A retro-auricular incision was made from posterior region of the earlobe along the retro-auricular sulcus (Fig. 1). Then it turned down and curved below the hair line [5, 8, 9]. The sub-platysmal skin flap was developed at level I to level III neck. The subcutaneous surgical tunnel was stabilized using the MODENA Retractor System [10], providing clear exposure of key anatomical boundaries under direct visualization with a headlight. These boundaries included the sternocleidomastoid (SCM) muscle posteriorly, the submandibular gland superiorly, the internal jugular vein anteriorly, and the omohyoid muscle inferiorly [8, 10] (Fig. 2). The great auricular nerve and accessory nerve were also identified and well-preserved during direct vision level II dissection [9].

**Fig. 3:**
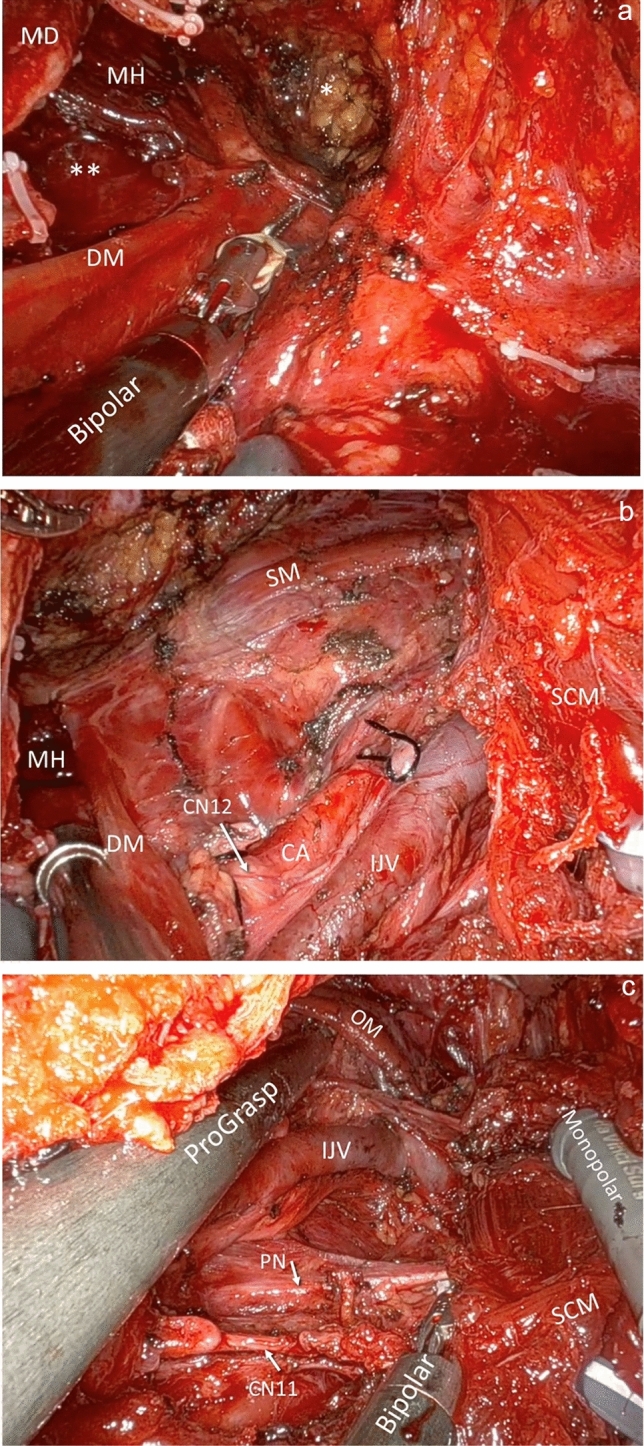
**a** Level Ia–Ib surgical field after completion of level I dissection. *Level Ia; **level Ib. *Bipolar* Maryland bipolar forceps, *DM* digastric muscle, *MD* mandible bone, *MH* mylohyoid muscle. **b** Level I–II surgical field after completion of level I–III dissections. *CA* carotid artery, *CN12* hypoglossal nerve, *DM* digastric muscle, *IJV* internal jugular vein, *MH* mylohyoid muscle, *SCM* sternocleidomastoid muscle, *SM* strap muscles. **c** Level II–III deep surgical field exposed by traction and counter-traction using three Endo-Wristed instruments, following completion of level I–III dissections. *Bipolar* Maryland bipolar forceps, *CN11* spinal accessory nerve, *IJV* internal jugular vein, *Monopolar* monopolar scissors, *PN* phrenic nerve, *OM* omohyoid muscle, *SCM* sternocleidomastoid muscle

**Fig. 4:**
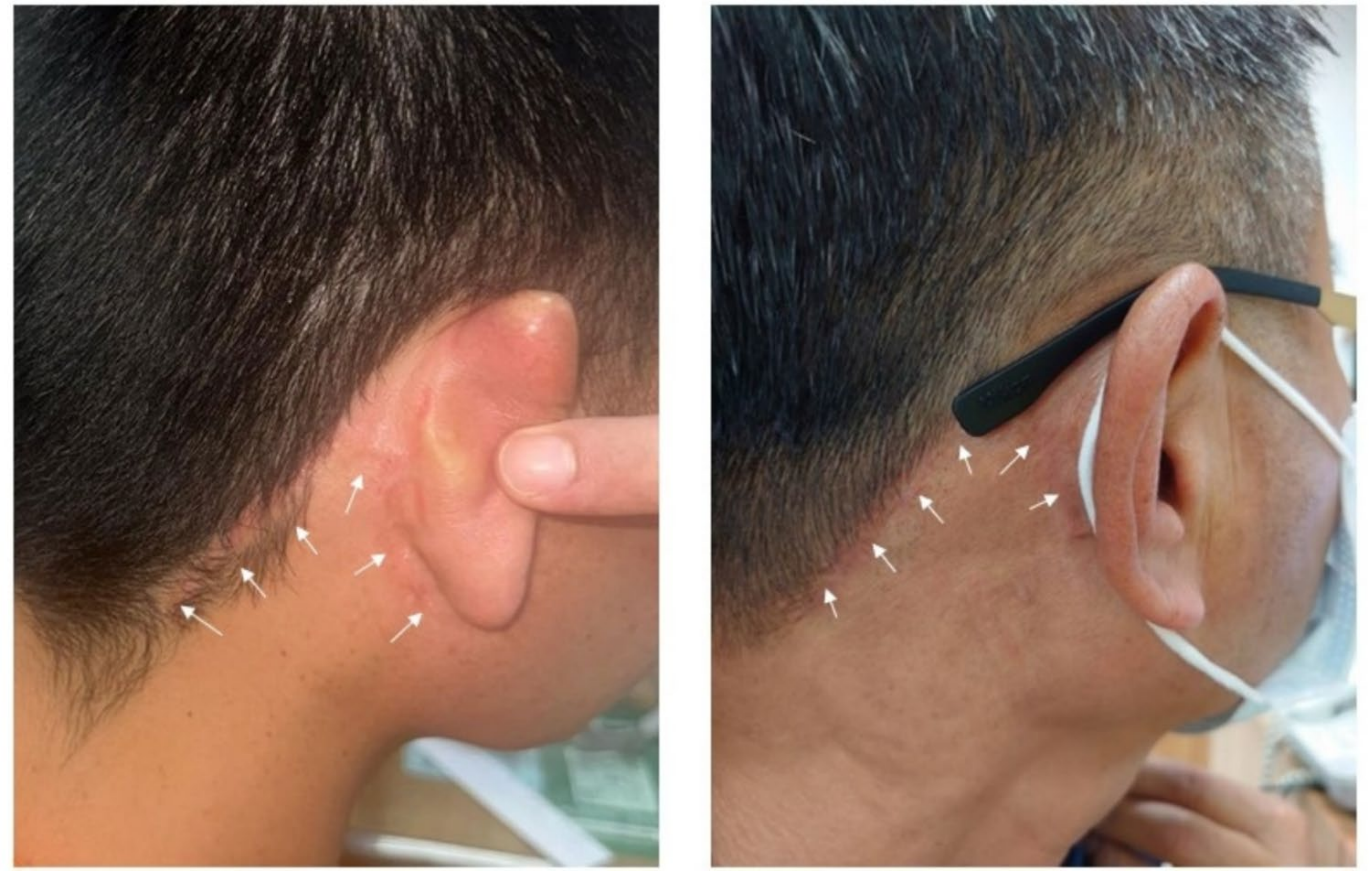
The retro-auricular surgical scars approximately 1 month after surgery in two different patients are shown with white arrows

Two or three robotic instruments, along with a 30-degree downward 3D high magnification endoscope of da Vinci robot, were inserted into the surgical tunnel through the retro-auricular incision. A Maryland dissector was placed at left hand side, while a monopolar curved scissors was employed as the right-hand instrument [5, 8] aside the 3D endoscopy. For the last five patients in our study group, an additional third instrument—ProGrasp forceps—was utilized to assist with traction and counter-traction. Meticulous dissection was commenced from level I. Following the completion of level I dissection, lympho-adipose tissue from levels II and III was carefully separated from the internal jugular vein, carotid sheath, and sternocleidomastoid muscle with preservation of vagus nerve and phrenic nerve similar to open surgery [5, 7, 8, 11]. Postoperative views of the surgical field are illustrated in Fig. 3a–c. After surgery, the scars are hidden behind the auricle, as demonstrated by the well-healed postoperative wounds of two different patients shown in Fig. 4.

### Outcome assessment

Perioperative outcomes including blood loss, hospital stay, duration of drainage tube placement, and postoperative complications, such as hematoma, infection, skin flap tip ischemia, and nerve injury, were obtained from the medical records. Oncological outcomes were assessed based on total lymph node yield, positive lymph nodes, adjuvant therapy, neck recurrence rates, and survival rates. These metrics were analyzed to compare the effectiveness and safety of the robotic and conventional approaches.

### Statistical analysis

Continuous variable were presented as median (IQR) and analyzed using the Mann–Whitney *U* test. Categorical variables, such as clinical nodal stage (cN), pathological nodal stage (pN), overall cancer stage, positive lymph nodes, adjuvant therapy, recurrence, and mortality, were analyzed using Fisher’s exact test. Statistical analyses were performed using the Statistical Package for the Social Sciences (SPSS), version 22.0 (IBM Corp., Armonk, NY, USA). A *p* value of < 0.05 was considered statistically significant.

## Results

The patients’ clinical characteristics and perioperative outcomes of the two groups are summarized in Table 1. There were no statistically significant differences between the two groups in terms of gender, primary cancer site, and clinical cancer stage. However, the median age was significantly younger in the robotic group (51 years) compared to the conventional group (61 years). In the robotic group, Da Vinci® Si system was used in 2 patients, while the Da Vinci® Xi system was used for the remaining 11 patients with 2 instruments (*n* = 8) or 3 instruments (*n* = 5). All robotic-assisted functional neck dissections were successfully completed without conversion to open neck dissection.

**Table 1:** Clinical characteristics of patients receiving robotic and conventional supraomohyoid neck dissection

	Total		Group				*p* value
			Robotic (*n* = 13)		Open (*n* = 13)		
	*n*	(%)	*n*	(%)	*n*	(%)	
Age, median(IQR)	55	(50.5–64.3)	51	(44–60)	61	(54–72)	0.014*
Gender							1.000
Male	20	(76.9%)	10	(76.9%)	10	(76.9%)	
Female	6	(23.1%)	3	(23.1%)	3	(23.1%)	
Primary site							0.051
Tongue	16	(61.5%)	9	(69.2%)	7	(53.8%)	
Buccal	7	(26.9%)	1	(7.7%)	6	(46.2%)	
RMT	2	(7.7%)	2	(15.4%)	0	(0%)	
Lip	1	(3.8%)	1	(7.7%)	0	(0%)	
Da Vinci type							–-
Si	2	(15.4%)	2	(15.4%)	0	(0%)	
Xi	11	(84.6%)	11	(84.6%)	0	(0%)	
Robotic instrument number							
Two	8	(61.5%)	8	(61.5%)	0	(0%)	
Three	5	(38.5%)	5	(38.5%)	0	(0%)	
cT stage							0.202
cT1	17	(65.4%)	10	(76.9%)	7	(53.8%)	
cT2	8	(30.8%)	2	(15.4%)	6	(46.2%)	
cT3	1	(3.8%)	1	(7.7%)	0	(0%)	
cN stage							1.000
cN0	24	(92.3%)	12	(92.3%)	12	(92.3%)	
cN1	1	(3.8%)	1	(7.7%)	0	(0%)	
cN2	1	(3.8%)	0	(0%)	1	(7.7%)	

Mann–Whitney test. Fisher’s exact test

*p < 0.05, **p < 0.01

**Fig. 5:**
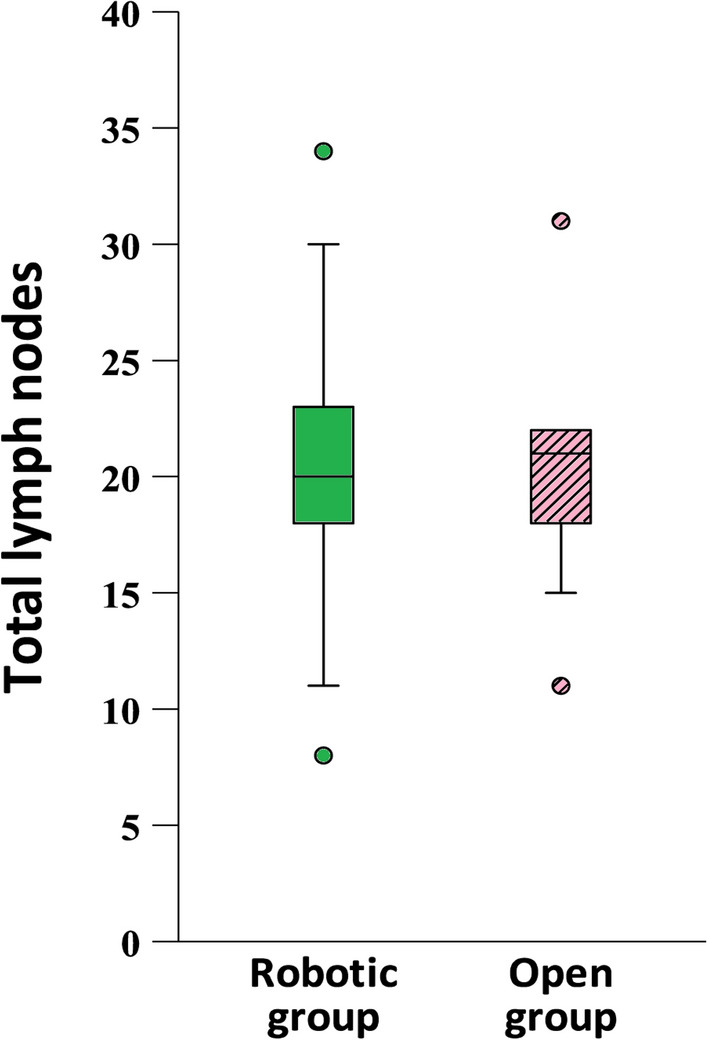
Box plot showing retrieved lymph nodes: the robotic group ranged from 8 to 34 (median 20), and the conventional group ranged from 11 to 40 (median 21)

Table 2 summarizes the postoperative and oncological outcomes. After median follow-up of 743 days, there were no significant differences between the two groups in terms of hospital stay, drainage duration, or complications. In the robotic group, two patients developed skin flap edge ischemia with minor infection, which was effectively managed with repeat aspiration and intravenous antibiotics. No cases of hypertrophic scarring or keloid formation were detected in either group during the follow-up period. No nerve injuries (accessory, hypoglossal, vagus, and phrenic) were observed in either group during follow-up. The robotic group had slightly fewer retrieved lymph nodes (range, 8–34; median, 20) than the conventional group (range, 11–40; median, 21) (Fig. 5), but the difference was not statistically significant (*p* = 0.950). Both groups had one patient with positive lymph nodes, resulting in a positive lymph node rate of 7.7% without extranodal extension. In terms of postoperative adjuvant therapy, two patients in each group received chemoradiation. No recurrences or patient deaths were observed during the follow-up period.

**Table 2:** Outcomes’ comparison between conventional and supraomohyoid neck dissection

	Total		Group				*p* value
			Robotic (*n* = 13)		Open (*n* = 13)		
	*n*	(%)	*n*	(%)	*n*	(%)	
Hospital stay(day), median(IQR)	7	(6–8.3)	6	(6–8)	7	(6–9)	0.491
Drainage duration (day), median (IQR)	4	(3–5)	4	(3–5)	4	(3–5)	1.000
Complication	2	(7.7%)	2	(15.4%)	0	(0%)	0.480
pT stage							0.467
pT1	17	(65.4%)	9	(69.2%)	8	(61.5%)	
pT2	5	(19.2%)	1	(7.7%)	4	(30.8%)	
pT3	3	(11.5%)	2	(15.4%)	1	(7.7%)	
pTx	1	(3.8%)	1	(7.7%)	0	(0%)	
pN stage							1.000
N0	24	(92.3%)	12	(92.3%)	12	(92.3%)	
N1	1	(3.8%)	1	(7.7%)	0	(0%)	
N2	1	(3.8%)	0	(0.0%)	1	(7.7%)	
Stage							0.156
I	16	(61.5%)	10	(76.9%)	6	(46.2%)	
II	6	(23.1%)	1	(7.7%)	5	(38.5%)	
III	3	(11.5%)	2	(15.4%)	1	(7.7%)	
IVa	1	(3.8%)	0	(0%)	1	(7.7%)	
Total lymph nodes, median(IQR)	20.5	(17.25–23.25)	20	(16–23.5)	21	(16.5–26.5)	0.950
Positive lymph nodes	2	(7.7%)	1	(7.7%)	1	(7.7%)	1.000
Adjuvant therapy	4	(15.4%)	2	(15.4%)	2	(15.4%)	1.000
Recurrence	0	(0%)	0	(0%)	0	(0%)	–
Death	0	(0%)	0	(0%)	0	(0%)	–
Follow-up time (day), median (IQR)	743	(214.8–1090.5)	353	(63.5–1058)	929	(589–1181)	0.081

Mann–Whitney test. Fisher’s exact test

*p < 0.05, **p < 0.01

In the robotic group, three instruments were used via da Vinci Xi platform in the last 5 out of 13 patients (Table 3). The median (IQR) operative time was 125.0 (110–156.5) min overall, with 121.0 (110–154.8) min in the two-instrument group and 131.0 (110–177) min in the three-instrument group (*p* = 0.803). The median lymph node yield was 20.0 (16–23.5) overall, with 20.5 (18.5–22.8) in the two-instrument group and 20.0 (9.5–29) in the three-instrument group (*p* = 0.831). There were no significant differences between the two groups.

**Table 3:** Comparison of robotic supraomohyoid neck dissection between 2 and 3 instruments

	Total (*n* = 13)		Instrument number 2 (*n* = 8)		Instrument number 3 (*n* = 5)		*p* value
	Median	IQR	Median	IQR	Median	IQR	
Operative time	125.0	(110–156.5)	121.0	(110–154.8)	131.0	(110–177)	0.803
Lymph node yield	20.0	(16–23.5)	20.5	(18.5–22.8)	20.0	(9.5–29)	0.831

Mann–Whitney *U* test

*p < 0.05, **p < 0.01

## Discussion

The risk of occult neck metastasis is around 15–20% in head and neck squamous cell carcinoma (HNSCC) patients, especially in oral cavity cancer patients [1]. While clinical N0 neck treatment options include elective neck dissection, chemoradiation (CRT), or observation [11], CRT often causes significant long-term toxicities affecting quality of life. Selective neck dissection involving levels I–III is recommended, as it aligns with typical nodal spread patterns in oral cavity cancer, providing accurate pathological staging and treating occult metastases [12]. However, the long transcervical skin incision of conventional open neck dissection from mid-neck to ipsilateral mastoid tip is frequently criticized for its esthetic impact, with hypertrophic scars or keloids imposing psychosocial and functional burdens [7, 12]. To avoid evident scar over neck, Kim et al. first introduced robotic-assisted lateral neck dissection via trans-axillary and retro-auricular (TARA) route for head and neck squamous cell carcinoma (HNSCC) since 2012 [5, 13]. Subsequent studies have consistently demonstrated its feasibility and safety, showing acceptable oncological outcomes with improved cosmetic satisfaction [5, 6, 8, 9, 12].

In our study, younger patients were more likely to opt for robotic-assisted surgery, possibly due to cosmetic concerns. Surgical outcomes such as hospital stay, drainage duration, and complications were comparable between the robotic and conventional groups. Minor skin flap ischemia occurred in two cases and resolved with simple management, consistent with a reported complication rate of approximately 3.2% [5], likely due to the skin flap traction during robotic procedures. In addition, although no hypertrophic scarring or keloid formation was observed at the retro-auricular incision site in the 13 patients, it can be speculated that patients prone to keloid formation may be particularly suitable for this procedure, as the resulting scar is hidden. No nerve injuries were identified after the dissections, underscoring the overall safety of the procedure.

The lymph node yield in the robotic group (median 20) was not significantly different from the open group (median 21), consistent with prior studies [9–11]. Of note, a lymph node yield ≥ 18 is associated with improved overall survival [14]. The positive lymph node rate in our study was 7.7%. Excluding clinical N-positive patients, only 1 out of 12 cN0 patients showed nodal metastasis on final postoperative histopathology, accounting for 9.2% in both the robotic and open groups. This rate is lower than previously reported [1], potentially reflecting advancements in preoperative imaging techniques that improve the correlation between clinical N0 and pathological N0 diagnoses. As the occult metastasis rate may not be as high as expected, more cN0 patients could be subjected to visible scarring from conventional surgery despite not requiring it. This underscores the advantage of robotic neck dissection—not only effectively managing potential occult metastasis but also concealing scars to maintain better cosmetic outcomes.

In the robotic group, three instruments were used in the last 5 out of 13 patients. Compared to the two-instrument approach, the addition of the ProGrasp forceps enhanced traction and counter-traction, improving tissue manipulation and surgical precision. While incorporating an additional instrument may require more preparation time, our results showed no significant increase in total operative time or lymph node yield. However, due to the limited number of cases, the statistical power may not be sufficient to draw definitive conclusions. As more cases are accumulated and surgical experience increases, we anticipate that the three-instrument technique may contribute to reduced operative time and improved lymph node retrieval in the future.

Over the past decade, numerous studies have highlighted the feasibility and benefits of robotic-assisted neck dissection in head and neck cancers but only two papers specifically focused on management of oral cavity cancers [11, 15] (Table 4). Studies such as those by Tae et al. and Park et al. have demonstrated that robotic-assisted neck dissection, particularly via postauricular and modified facelift approaches, achieves oncological outcomes comparable to conventional methods while offering superior cosmetic results [6, 8]. Dabas et al. further expanded the repertoire of robotic techniques by introducing the modified BABA approach, showcasing its versatility in diverse clinical scenarios [16]. Our study builds on this foundation by focusing specifically on oral cancer, allowing for more precise treatment planning. In addition, we documented advancements in surgical techniques at our institution, including the transition from the Si to Xi da Vinci systems and the adoption of a third robotic instruments to facilitate traction and counter-traction during the surgery.

**Table 4:** Clinical characteristics of studies on robotic neck dissection for head and neck cancers

Study (year)	Kim 2012 [13]	Lee 2012 [11]	Park 2013 [6]	Albergotti 2014 [9]	Tae 2014 [8]	Poma 2022 [17]	Kim 2024 [15]	Dabas 2024 [16]
Sample size *n*								
Robotic	7	10	7	3	11	10	32	82
Open	0	16	0	6	19	5	46	0
Study type	P	R	P	R	R	R	R	P
Primary site (robotic)	OC, Opx, Hpx	OC	Opx, Hpx	Opx	OC, Opx, Larynx	OC, Opx, Larynx	OC	OC, Opx, Hpx, CUP
Clinical N-status	cN0 and cN+	cN0 only	cN0 only	cN0 and cN+	cN0 only	cN0 and cN+	cN0 only	cN0 and cN+
Da Vinci system	S	S	NR	NR	NR	NR	NR	Xi
Neck approach	TARA	MFL, RA	MFL, RA	MFL	RA	TARA, RA	RA	BABA
Arms number*	3	2	2	2	2	NR, 3	3	3
Lymph node yield								
Robotic	36	22	22	33.3	25	29.4, 28.6	19.22	28.2
Open	N/A	20	N/A	24.8	28.9	26.6	20.7	N/A
Country	SK	SK	SK	USA	SK	Italy	SK	India

*Endoscope arm not counted in the total arm number

*BABA* bilateral axillo-breast insufflation, *CUP* carcinoma of unknown primary, *HPx* hypopharynx cancer, *MFL* modified face lift, *N/A* not applicable, *OC* oral cavity cancer, *OPx* oropharynx cancer, *NR* no reported, *P* prospective, *PTC* papillary thyroid cancer, *R* retrospective, *RA* retro-auricular, *SK* South Korea, *SND* selective neck dissection, *TARA* trans-axillary retro-auricular, *USA* United States of America

Our study has several important limitations. First, operative time and perioperative blood loss could not be directly compared because most patients in the open surgery group underwent simultaneous primary tumor excision and neck dissection, while the robotic group received neck dissection alone. Second, this was a retrospective, single-center study, which may introduce selection bias, particularly because robotic surgery was self-paid. Patients with higher income or stronger cosmetic concerns may have been more likely to choose the robotic approach, which could have biased the patient cohort and affected cosmetic outcome perceptions. These factors substantially restrict the generalizability of our findings, which should, therefore, be interpreted as preliminary. Further multi-center studies with larger cohorts and longer follow-up are warranted to confirm the oncological safety and cosmetic benefits of robotic-assisted neck dissection.

In conclusion, our study results indicate that robot-assisted supraomohyoid neck dissection via a retro-auricular approach is technically acceptable and oncological feasible for clinical N0 oral cavity cancer neck management. In da Vinci Xi platform, three instruments could also be used to obtain better traction and counter-traction during dissection. The robotic surgery provides an esthetically superior alternative without compromising surgical or oncological outcomes. This technique offers early stage oral cavity cancer patients, particularly those with clinical N0 disease, an additional motivation to undergo prophylactic neck dissection. Future studies with larger cohorts and extended follow-up are warranted to confirm the oncological safety and cosmetic advantages of robotic-assisted supraomohyoid neck dissection compared to conventional methods.

## Data availability statement

Data are available upon reasonable request to the corresponding author.

## Declarations

### Permissions statement

No copyrighted material from other sources requiring permission was used in this manuscript.
